# Effects of *Gynura procumbens* extract supplementation on growth performance, carcass traits, antioxidant capacity, immunity and meat quality of meat ducks

**DOI:** 10.3389/fvets.2024.1508048

**Published:** 2024-12-13

**Authors:** Gaoxiang Ai, Pingwen Xiong, Jiang Chen, Wenjing Song, Qiongli Song, Chuanhui Xu, Weide Su, Zhiheng Zou, Qipeng Wei, Xiaolian Chen

**Affiliations:** ^1^Institute of Animal Husbandry and Veterinary Science, Jiangxi Academy of Agricultural Sciences, Nanchang, China; ^2^Jiangxi Province Key Laboratory of Animal Green and Healthy Breeding, Nanchang, China

**Keywords:** *Gynura procumbens* extract, immunity, antioxidant capacity, meat quality, meat ducks

## Abstract

**Introduction:**

*Gynura procumbens* (Lour.) Merr is a common traditional Chinese medicine with anti-tumor, anti-inflammatory and antioxidant activities. However, no related studies reported the potential application effect of *Gynura procumbens* on meat ducks. The study aims to investigate the potential effects of *Gynura procumbens* extract (GPE) supplementation on growth performance, carcass traits, antioxidant capacity, immunity and meat quality.

**Methods:**

A total of 480 21-day-old female healthy ducks were randomly allocated to four treatments, each treatment containing six replicates with 20 ducks per replicate. The groups received a corn-soybean basal diet supplemented with 0 mg/kg GPE (CON), 200 mg/kg GPE (GPE200), 400 mg/kg GPE (GPE400), and 600 mg/kg GPE (GPE600), respectively. The entire experiment lasted for 7 weeks.

**Results:**

The results showed that dietary supplementation with 600 mg/kg GPE significantly reduced the contents of serum urea nitrogen, triglyceride (TG) and total cholesterol (TC). GPE (200, 400, and 600 mg/kg) supplementation effectively reduced the contents of IL-2 and MDA. The levels of immunoglobulin M (IgM) as well as total antioxidative capacity (T-AOC) in GPE600 group dramatically elevated in comparison with the control group. Dietary GPE supplementation considerably increased the moisture content of the breast muscle. Furthermore, dietary supplementation with GPE markedly decreased the water loss rate and shear force.

**Discussion:**

With the ban of antibiotics in poultry production, traditional Chinese medicines have been widely used in livestock and poultry production due to their high efficiency and low toxicity. *Gynura procumbens* extract GPE as a natural plant origin contains a series of biologically active components, including flavonoids, polyphenols, saponin, tannin and terpenoid. This study indicated that dietary supplementation with GPE can increase serum total antioxidant capacity, regulate immune function and improve meat quality to some extent in meat ducks. The recommended optimal GPE level in the diet of meat ducks is 600 mg/kg according to the results in this study.

## 1 Introduction

With the advent of biological and engineering technology, large numbers of new antibiotics were developed to treat diseases. In recent years, various antibiotics have been regularly used in medical treatment as well as livestock farming. The application of antibiotics in feed has greatly improved the growth performance of animals and promoted the rapid development of animal husbandry (1). Unfortunately, the extensive abuse of antibiotics resulted in significant antibiotic residues in meat and aquatic products, as well as increased the risks of drug-resistant strains mutations (2). Meanwhile, with the improvement in living standards, consumers have a stronger desire to pursue high quality natural foods. However, long-term misuse of antibiotics can undermine the original intention of ecological farming and simultaneously pose a substantial threat to public health (3–6). Therefore, there is an urgent need to search for alternative novel additives to antibiotics.

It is widely believed that nutritional regulation offers an effective strategy to replace antibiotics through the use of traditional Chinese medicine (TCM) additives. As the treasures of the Chinese nation, Chinese herbal medicines has a long history of being widely used in the treatment of various diseases among the people (7). Recently, traditional Chinese herbs have attracted increasing attention from agronomists due to their high efficacy and low toxicity. As we all know, with the gradual ban of antibiotics in livestock farming, more and more researchers have turned their attention toward the development of Chinese herbal additives. Indeed, many medicinal plants and bioactive compounds originated from natural plants have been widely used in livestock farming (8) For instance, Curcumin, a polyphenolic curcuminoid compound derived from the rhizomes of the plant *Curcuma longa*, has been used as feed additive for improving the intestinal health of broiler (9). Traditional Chinese medicinal herbs prescription composed of Cortex Fraxini, Pulsatilla chinensis and *Eucommia ulmoides* could improve ducks’ antioxidant and immune capacity (10).

As a common folk TCM, GP (*Gynura procumbens (Lour.) Merr.*) is an annual evergreen shrub with a fleshly stem and purple tint and is considered to possess diverse pharmacological properties, such as antioxidation, antihypertension and cardioprotective, anti-inflammatory, antihyperglycemic and anticancer activities (11). It has been extensively used in clinic as remedy for eruptive fevers, rashes, kidney diseases, hypertension, diabetes mellitus and cancer (12, 13). These beneficial pharmacological activities of GP may be attributed to the presence of its active ingredients, including flavonoids, saponin, tannin, terpenoids and glycosides (14, 15). Currently, vast attention has been focused on the broad clinical application of GPE. However, no studies are exploring the potential effects of GPE supplementation on ducks.

Currently, in the process of duck farming, Low immunity among duck flocks remains a pressing challenge for farmers and is widely recognized as a key factor contributing to high duck mortality rates. Additionally, ducks are highly susceptible to stress responses triggered by high-density farming, which results in oxidative damage and further causes the reduction of growth performance (16). Therefore, maintaining the balance of the oxidant/antioxidant and enhancing immunity is an effective approach to promote the health growth of ducks and plays a fundamental role in improving the quality of duck meat (17). Numerous clinical studies highlight that GPE is abundant in polyphenols and flavonoids, which plays an important role in boosting immunity and strengthening antioxidant defenses (18, 19). Despite GPE’s widespread clinical applications, no related studies to date have explored its potential effects on ducks. Our preliminary study has indicated that dietary supplementation with GPE could boost duck’s immunity. Therefore, the current study was designed to explore the potential effect of dietary supplementation with GP on growth performance, immunity and antioxidative capacity of ducks and further elucidate the important role of GPE in improving meat quality.

## 2 Materials and methods

### 2.1 Animals and experimental design

**Figure 1:**
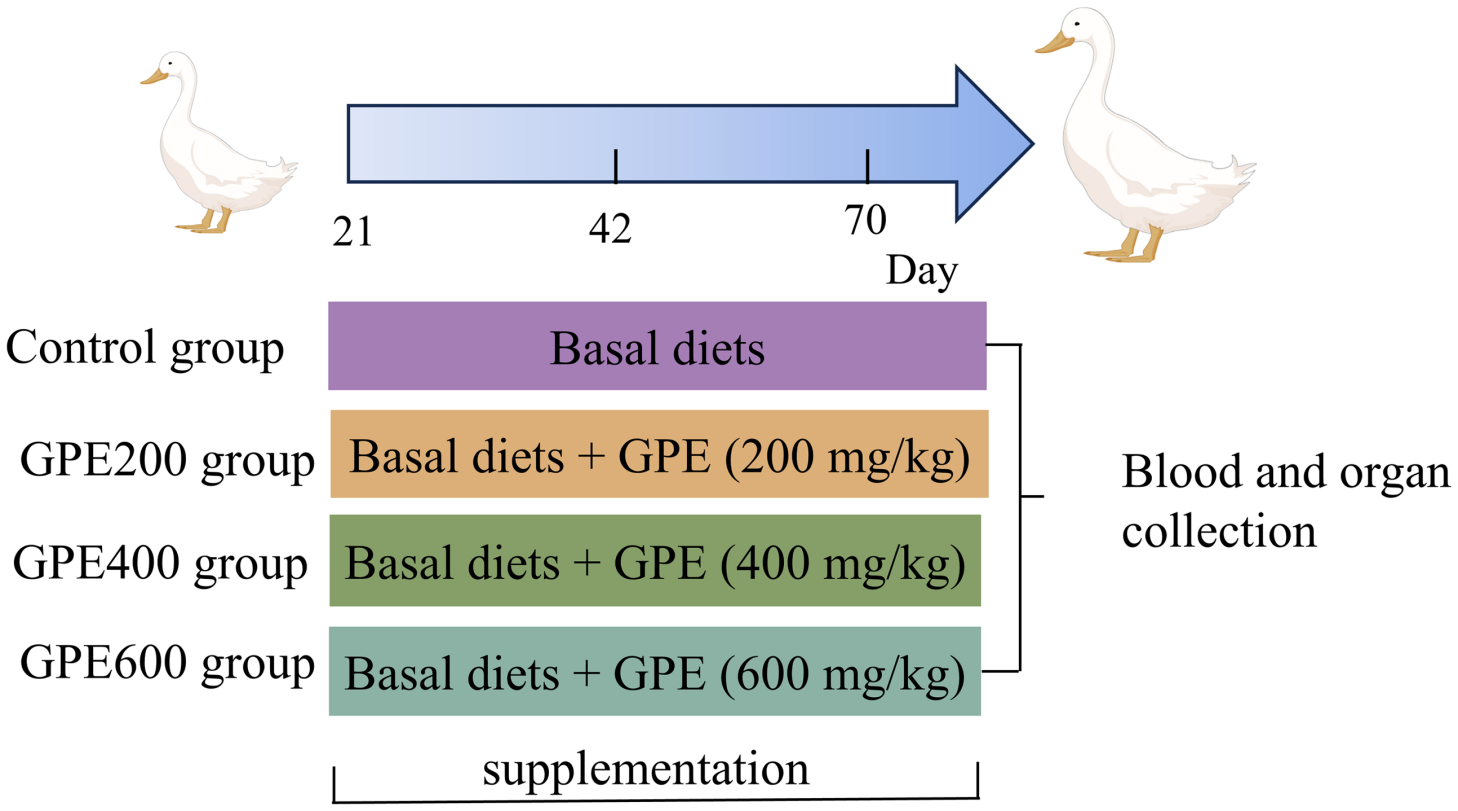
Schematic diagram of experiment protocol.

The experiment was carried out with permission from the Experimental Animal Ethics Committee in Jiangxi Academy of Agricultural Sciences. A total of 480 21-day-old female healthy ducks with similar body weight were bought from a local commercial hatchery (Jian, China). Subsequently, they were randomly assigned into four treatments with six replicates each comprising 20 ducks. The four dietary treatments consisted of the control and three levels of GPE (200 mg/kg, 400 mg/kg, and 600 mg/kg). The basal diet was formulated to meet the nutrient requirements of meat ducks (NY/T 2122–2012, China). The ingredient compositions and nutrient contents of the basal diets for ducks are listed in Table 1. These ducks were rearing on net bed with splash pool and received water *ad libitum* through the entire experiment period. The whole experiment was sustained for 7 weeks, the schematic diagram showing the study design is displayed in Figure 1.

**Table 1:** Composition and nutrient levels of the basal diet (%, as fed basis).

Items	Day 21–42	Day 43–70
**Ingredients (%)**		
Corn	63.82	71.01
Soybean meal	24.02	19.05
Wheat middling and reddog	8.00	6.00
*Moringa oleifera* leaf	0.00	0.00
Soybean oil	0.00	0.00
*L*-Lysine•HCl	0.02	0.00
*DL-Methionine*	0.17	0.12
Limestone	1.17	1.18
Dicalcium phosphate	1.28	1.28
Salt (NaCl)	0.35	0.36
Premix^a^	1.00	1.00
Total calculated nutrient levels	100	100
Metabolizable energy (MJ/kg)	2.87	2.92
Crude protein (%)	17.00	15.00
Lysine (%)	0.85	0.71
Methionine (%)	0.42	0.35
Calcium (%)	0.85	0.80
Available phosphorous (%)	0.60	0.35
Methionine + Cysteine (%)	0.70	0.60

a The premix provides the following per kg of diets: vitamin A, 11,500 IU; vitamin D3, 33,000 IU; vitamin E, 25 IU; vitamin K3, 3 mg; vitamin B1, 2 mg; vitamin B2, 8 mg; vitamin B6, 4.5 mg; vitamin B12, 0.03 mg; D-pantothenic acid, 12 mg; nicotinic acid, 50 mg; choline chloride, 1,000 mg; biotin, 0.2 mg; folic acid, 0.6 mg; antioxidant, 100 mg; Fe, 60 mg; Cu, 8 mg; Mn, 90 mg; Zn, 60 mg; I, 0.4 mg and Se, 0.2 mg.

### 2.2 Growth performance

After 12 h of fasting, the body weight (BW) from each replicate cage was measured on days 21, 42, and 70. Mortality was monitored daily, with the weight of dead ducks recorded and feed intake per cage was recorded every day through the experimental period. Subsequently, the average body weight, average daily feed intake, average daily gain, and the ratio of feed to weight gain (F/G) were calculated with correction for mortalities.

### 2.3 Blood sample collection

Two ducks from each replicate were randomly selected at 42 and 70 days of age, respectively. Blood was obtained from wing vein and centrifugated at 3,000 g, for 10 min at 4°C to prepare serum for subsequent measurement.

### 2.4 Measurement of serum biochemical indices, antioxidant activity and immunity

For biochemical assays, the freshly collected serum samples were used to measure the activities of total antioxidant capacity (T-AOC, Cat no, A015-2-1), glutathione peroxidase (GSH-Px, Cat no, A005-1-2), superoxide dismutase (SOD, Cat no, A001-3-2), malondialdehyde (MDA, Cat no, A003-1-2), blood urea nitrogen (BUN, Cat no, C013-2-1), alkaline phosphatase (ALP, Cat no, A059-2-2), aspartate aminotransferase (AST, Cat no, C010-2-1), alanine aminotransferase (ALT, Cat no, C009-2-1), triglyceride (TG, Cat no, A110-2-1) and albumin (ALB, Cat no, A028-2-1) using corresponding commercial assay kits (Jiancheng Bioengineering, Nanjing, China). The contents of immunoglobulin A (IgA, Cat no, ml036901), immunoglobulin G (IgG, Cat no, ml036900) and immunoglobulin M (IgM, Cat no, ml061224) were quantified with ELISA assay kits (Shanghai Enzyme-linked Biotechnology, China) according to the manufacturer’s instructions. Additionally, sample quality control and testing parameter setting were strictly adhered to the product specification.

### 2.5 Carcass traits and organ indices

At the end of the trial (70 days of age), two ducks from each replicate were randomly selected, weighed individually and sacrificed after 12 h of feed deprivation. Subsequently, ducks were sacrificed by exsanguination from the jugular vein. The weight of the defeather carcass was determined as the carcass weight. The carcass was weighed after removing the trachea, esophagus, spleen, pancreas, gallbladder, reproductive organ, intestinal tract, gizzard content and gizzard-membrane which was recorded as semi-eviscerated weight. The eviscerated weight was measured as eviscerated weight after removing the heart, liver, lung, gizzard, glandular stomach, and abdominal fat. Finally, the rate of dressing, semi-eviscerated, eviscerated, breast muscle, leg muscle, and abdominal fat were calculated. The weight of dissected heart, liver, spleen, lung, gizzard and glandular stomach was recorded, respectively, and organ index was calculated.

### 2.6 Evaluation of liver histopathological changes

For histopathological analysis, liver tissues of ducks were collected in ice-cold phosphate-buffered saline and subsequently transferred into 4% fresh paraformaldehyde for fixation at room temperature for at least 24 h. Subsequently, the specimens were further processed for dehydration. After dehydration with ethanol and xylene, the tissues were embedded in paraffin, sectioned at a thickness of 5 μm and subjected to hematoxylin and eosin (H&E). Afterward, the above samples were photographed under a light microscope (BX53, Olympus).

### 2.7 Meat quality

After slaughter, the pH values of breast muscle and leg muscle were measured with a portable pH meter (pH-Star, Matthaus GmbH & Co. KG, Eckelsheim, Germany) in the pectoralis major muscle and leg muscle at about 1 cm depth as Huang previously described (20). Color changes were evaluated at each sampling by measuring L^*^, a^*^, and b^*^ parameters using a reflectance spectrophotometer (X-Rite SP64, United States), L^*^, a^*^, and b^*^ values represented the degree of lightness, greenness or redness, and blueness or yellowness, respectively. Subsequently, the shear force was measured. The procedure was as follows: the raw breast muscles were cut into three strips (1 × 1 × 3 cm) perpendicular to the muscle fiber direction to evaluate the tenderness using a digital-display muscle tenderness meter (C-LM3B, Tenova, Harbin, China). The water loss rate was measured according to the modified method of Li et al. (21). In brief, collected meat samples (0.125 cm^3^) were weighed and carefully wrapped in absorbent and placed into the machine for testing. The program was set to 300 N for 5 min. After 5 min, the samples were weighed again. Finally, the water loss rate was calculated as a percentage:


$$\begin{array}{l} \mathrm{Water loss rate}=\mathrm{[(initial muscle weight}\;-\;\mathrm{final muscle weight) /}\\ \mathrm{initial muscle weight]}*100\%\text{.} \end{array}$$

### 2.8 Determination of IMP content in muscle

The inosine Monophosphate (IMP) concentration was determined with high-performance liquid chromatography (HPLC, Agilent 1290, Agilent Technologies, United States). The IMP content in the samples was measured using an acidic extraction method on approximately 1 g of dry weight samples. In brief, 25 mL of phosphoric acid was first added to the sample. Subsequently, it was gently mixed and incubated on ice for 30 min with intermittent gentle shaking. After extraction for 30 min in ice-water bath, the supernatant was collected after centrifugation (3,000 rpm/min, 5 min) and then transferred to a clean 50 mL centrifuge tube. Immediately afterward, the supernatant was adjusted to pH 6.5 with 5 M sodium hydroxide solution and topped to 50 mL with the additional supernatant. Afterward, the obtained solution was filtered through 0.45 μm organic filter membrane before being used for the HPLC analysis. Finally, the content of IMP in the filtrate was analyzed by HPLC. The HPLC conditions were as described above: column: Phenomenex Luna C18 100 Å column (250 mm × 4.6 mm, 5 μm); mobile phase: 0.05 M triethylammonium dihydrogen phosphate/acetonitrile (95/5); flow rate: 1 mL/min; detection wavelength: 254 nm; column temperature: 25°C.

### 2.9 Chemical composition of the duck breast muscle

Protein, fat and moisture contents in duck breast muscle (stored at 4°C) were determined following the guidelines from the Associate of Analytical Chemists.

### 2.10 Analysis of antioxidative enzyme activities in duck breast muscle

Duck breast muscle (100 mg, stored in air 4°C for 24 h) was accurately weighed and added with ice-cold phosphate buffer solution (4°C, 0.01 mol/L, pH = 7.2–7.4) to prepare 10% w/v homogenate. Firstly, the protein content of 10% homogenate was measured by bicinchoninic acid (BCA) protein assay reagent. Subsequently, the activities of SOD (Cat no, A001-3-2), GSH-Px (Cat no, A005-1-2), T-AOC (Cat no, A015-2-1), total cholesterol (TC, Cat no, A111-2-1) and the concentration of MDA (Cat no, A003-1-2) were determined using the commercial kits (Nanjing Jiancheng Biology Engineering Institute, Nanjing, China) following the instructions of the manufacturer, respectively.

### 2.11 Statistical analysis

All results are presented as the means ± (SEM) standard errors of the means. The experimental data were statistically analyzed using the SPSS version 19.0 software (SPSS Inc., Chicago, IL, United States). For the comparison between two experiment groups, statistical significance was assessed using Student’s *t*-test. For multiple groups, the statistical significance of the differences was carried out by one-way analysis followed by Turkey test. Differences between groups were considered statistically significant for *p* < 0.05.

## 3 Results

### 3.1 Growth performance

As showcased in Table 2, in comparison to the control group, there was no significant difference in body weight, average daily feed intake (ADFI) and average daily gain (ADG) with increasing levels of GPE throughout the experimental period. Additionally, there was no statistically considerable variation in F/G between the control and the treatment groups.

**Table 2:** Effect of GPE supplementation on growth performance of meat ducks.

Items^a^	Time	Diets				SEM	*p*
		CON	GPE200	GPE400	GPE600		
BW/g	Day 21	421.0	421.0	421.1	420.9	0.06	1.000
	Day 42	1282.34	1282.13	1282.10	1279.39	6.99	0.999
	Day 70	1943.20	1892.00	1937.00	1895.80	18.27	0.690
ADG/(g/d)	Day 21–42	40.29	41.04	41.00	40.78	0.31	0.843
	Day 43–70	23.03	21.52	22.92	20.96	0.51	0.414
	Day 21–70	30.43	29.89	30.67	29.45	0.36	0.670
ADFI/(g/d)	Day 21–42	123.38	123.98	124.49	123.89	1.13	0.991
	Day 43–70	151.09	148.88	151.58	148.05	2.01	0.925
	Day 21–70	139.22	138.21	139.97	137.69	1.44	0.955
F/G	Day 21–42	3.06	3.02	3.04	3.04	0.02	0.941
	Day 43–70	6.58	6.96	6.65	7.07	0.09	0.184
	Day 21–70	4.57	4.63	4.57	4.68	0.03	0.566

BW, ADG, ADFI, and F/G represent the means of 6 replicates. BW, body weight; ADG, average daily gain; ADFI, average daily feed intake; F/G, ratio of feed to weight gain; SEM, standard error (SE) of means.

a Data are the mean of 6 replicates with 20 ducks each replicate.

### 3.2 Serum biochemical parameters

To probe whether GPE exerted potential protective effect on antioxidation, lipid metabolism and liver function, related biochemical indexes were measured by biochemical kits. The results are presented in Table 3. Relative to the control group, dietary GPE supplementation prominently (all for *p* < 0.05) decreased the activities of BUN and TG at 42 and 70 days of age. Moreover, dietary GPE inclusion significantly elevated the total protein level. Notably, at 42 and 70 days of age, as the concentration of GPE increased, BUN and TG activities progressively decreased, reaching their lowest at a dietary concentration of 600 mg/kg GPE. However, supplementation with GPE had no discernible effect on the contents of ALP, AST, ALT and ALB (all for *p* > 0.05).

**Table 3:** Effect of GPE supplementation on serum biochemical parameters of meat ducks.

Items^1^	Diets				SEM	*p*
	CON	GPE200	GPE400	GPE600		
Day 42						
Total protein (g/L^−1^)	28.41^c^	29.13^bc^	30.87^a^	30.26^ab^	0.30	0.012
ALB (g/L^−1^)	12.78	12.99	13.31	12.95	0.18	0.779
BUN (μmol∙L^−1^)	388.29^a^	378.59^ab^	365.39^b^	346.78^c^	3.71	<0.001
TG (mmol∙L^−1^)	2.60^a^	2.46^ab^	2.38^b^	2.32^b^	0.03	0.019
TC (mmol∙L^−1^)	2.71	2.56	2.49	2.51	0.03	0.084
ALP (U∙L^−1^)	5504.30	5521.69	5543.72	5555.57	25.05	0.899
AST (U∙L^−1^)	126.62	126.88	127.13	127.19	0.99	0.997
ALT (U∙L^−1^)	2.34	2.33	2.38	2.37	0.03	0.923
**Day 70**						
Total protein (g/L^−1^)	28.82^b^	30.67^ab^	31.78^a^	31.64^a^	0.38	0.018
ALB (g/L^−1^)	12.62	13.04	13.48	13.36	0.13	0.084
BUN (μmol∙L^−1^)	381.03^a^	361.11^b^	361.44^b^	343.40^c^	2.91	<0.001
TG (mmol∙L^−1^)	2.69^a^	2.33^b^	2.29^b^	2.25^b^	0.04	<0.001
TC (mmol∙L^−1^)	2.80^a^	2.46^b^	2.36^b^	2.34^b^	0.04	<0.001
ALP (U∙L^−1^)	5507.99	5515.43	5520.27	5533.74	24.43	0.987

SEM, standard error (SE) of means.

1 Different lowercase letters represent significant differences (*p* < 0.05) and data are the mean of 6 replicates with 2 ducks each replicate.

### 3.3 Serum immunity and inflammatory parameters

To further interrogate the effect of GPE on cellular immunity, the ELISA analysis was used to detect the concentrations of serum IL-6, IL-1β, IgA, IgM, and IgG. As depicted in Table 4, with respect to the control group, the IgM level was markedly (*p* < 0.01) enhanced at 42 and 70 days of age after dietary supplementation with GPE. Additionally, at 42 days of age, supplementation with GPE had no obvious effect on IgG activity. However, supplementation with GPE apparently elevated the IgG and IgM activities at 70 days of age. Furthermore, the concentration of IL-2 was notably lowered in GPE-treated groups at 42 and 70 days of age compared with the control group. Of note, there are no significant differences observed in IL-1β, IL-6, and IgA. These results demonstrated that dietary supplementation with GPE could improve immune function by enhancing the activities of immune factors and modulating the levels of inflammatory cytokines.

**Table 4:** Effect of GPE supplementation on serum immunity related indicators of meat ducks.

Items^1^	Diets				SEM	*p*
	CON	GPE200	GPE400	GPE600		
**Day 42**						
IgA (g∙L^−1^)	2.15	2.27	2.29	2.31	0.03	0.117
IgG (g∙L^−1^)	3.83	3.94	4.04	3.91	0.03	0.051
IgM (g∙L^−1^)	1.41^b^	1.67^a^	1.73^a^	1.76^a^	0.03	<0.001
IL-1β (mg∙mL^−1^)	0.20	0.20	0.21	0.21	0.00	0.057
IL-6 (mg∙mL^−1^)	0.21	0.19	0.18	0.18	0.01	0.457
IL-2 (mg∙mL^−1^)	3.17^a^	2.81^b^	2.75^b^	2.72^b^	0.05	0.002
**Day 70**						
IgA (g∙L^−1^)	2.16	2.25	2.27	2.31	0.02	0.156
IgG (g∙L^−1^)	3.84^b^	3.90^ab^	4.05^a^	4.05^a^	0.03	0.029
IgM (g∙L^−1^)	1.42^b^	1.73^a^	1.76^a^	1.77^a^	0.03	<0.001
IL-1β (mg∙mL^−1^)	0.21	0.21	0.21	0.21	0.00	0.733
IL-6 (mg∙mL^−1^)	0.20	0.19	0.19	0.18	0.00	0.439
IL-2 (mg∙mL^−1^)	3.12^a^	2.84^b^	2.77^b^	2.70^b^	0.04	<0.001

SEM, standard error (SE) of means.

1 Different lowercase letters represent significant differences (*p* < 0.05) and data are the mean of 6 replicates with 2 ducks each replicate.

### 3.4 Serum antioxidant status

Antioxidant activity refers to the ability to eliminate free radicals in the body. To evaluate the antioxidant potential of GPE, the levels of oxidative stress-related indicators (T-AOC, SOD, MDA, and GSH-Px) were detected by corresponding assay kits. As displayed in Table 5, the level of T-AOC was distinctly elevated at 42 and 70 days of age following GPE supplementation, showing a dose-dependent manner with higher concentrations of GPE. Furthermore, in contrast to the control group, at 70 days of age, dietary supplementation with GPE conspicuously reduced MDA level, with 600 mg/kg GPE treatment showing the lowest levels. However, no prominent changes are observed in SOD and GSH-Px throughout the duration of the experiment after dietary supplementation with GPE.

**Table 5:** Effect of GPE supplementation on serum anti-oxidant related indicators of meat ducks.

Items^1^	Diets				SEM	*p*
	CON	GPE200	GPE400	GPE600		
**Day 42**						
T-AOC (U∙L^−1^)	3.33^c^	3.56^bc^	3.72^a^	3.60^ab^	0.03	0.000
SOD (U∙L^−1^)	108.35	109.66	111.16	111.67	1.03	0.672
GSH-Px (U∙L^−1^)	1.29	1.34	1.35	1.34	0.02	0.611
MDA (μmol∙L^−1^)	8.28	7.96	8.07	7.98	0.08	0.556
**Day 70**						
T-AOC (U∙L^−1^)	3.53^b^	3.60^b^	3.77^a^	3.82^a^	0.03	0.003
SOD (U∙L^−1^)	114.61	116.05	117.68	117.01	1.52	0.908
GSH-Px (U∙L^−1^)	1.15	1.19	1.24	1.21	0.02	0.162
MDA (μmol∙L^−1^)	7.98^a^	7.68^b^	7.62^b^	7.64^b^	0.05	0.039

SEM, standard error (SE) of means.

1 Different lowercase letters represent significant differences (*p* < 0.05) and data are the mean of 6 replicates with 2 ducks each replicate.

### 3.5 Carcass traits

The effect of GPE supplementation on carcass traits of ducks were displayed in Table 6. Compared to the control group, all treatments did not show any significant effects on semi-eviscerated yield, dressed yield, eviscerated yield, breast muscle yield, leg muscle yield and abdominal fat yield. Although there were no differences in these indicators, it was worth noting that dietary supplementation with GPE decreased abdominal fat, especially 600 mg/kg.

**Table 6:** Effect of GPE supplementation on carcass traits of meat ducks.

Items^1^	Diets				SEM	*p*
	CON	GPE200	GPE400	GPE600		
Dressed yield, %	90.11	89.88	89.68	89.36	0.17	0.451
Semi-eviscerated yield, %	83.27	82.89	83.07	82.51	0.19	0.544
Eviscerated yield, %	74.89	74.87	74.32	73.88	0.25	0.422
Breast muscle yield, %	13.38	13.51	12.54	13.25	0.21	0.413
Leg muscle yield, %	11.45	11.17	10.58	10.94	0.12	0.086
Abdominal fat yield, %	2.46	2.20	2.17	1.98	0.09	0.288

SEM, standard error (SE) of means.

1 Data are the mean of 6 replicates with 2 ducks each replicate.

### 3.6 Organ indexes

The effects of GPE on relative weights of liver, kidney, spleen, lung, gizzard and glandular stomach are illustrated in Table 7. Moreover, the kidney, spleen, lung, gizzard and glandular stomach weight were no obvious differences between the control group and the different dosages of GPE-treated groups. These results revealed that GPE had no obvious toxic and side effects to major organs *in vivo.*

**Table 7:** Effect of GPE supplementation on organ index of meat ducks.

Items^1^	Diets				SEM	*p*
	CON	GPE200	GPE400	GPE600		
Heart, g/kg	7.57	7.65	7.71	7.94	0.14	0.819
Liver, g/kg	19.22	19.81	19.50	19.06	0.41	0.928
Spleen, g/kg	0.73	0.69	0.89	0.70	0.04	0.255
Lung, g/kg	9.45	9.89	9.02	10.43	0.28	0.338
Glandular stomach, g/kg	3.65	3.56	3.73	3.70	0.11	0.955
Gizzard, g/kg	24.92	26.68	26.19	27.72	0.60	0.421

SEM, standard error (SE) of means.

1 Data are the mean of 6 replicates with 2 ducks each replicate.

### 3.7 Hepatic morphology

**Figure 2:**
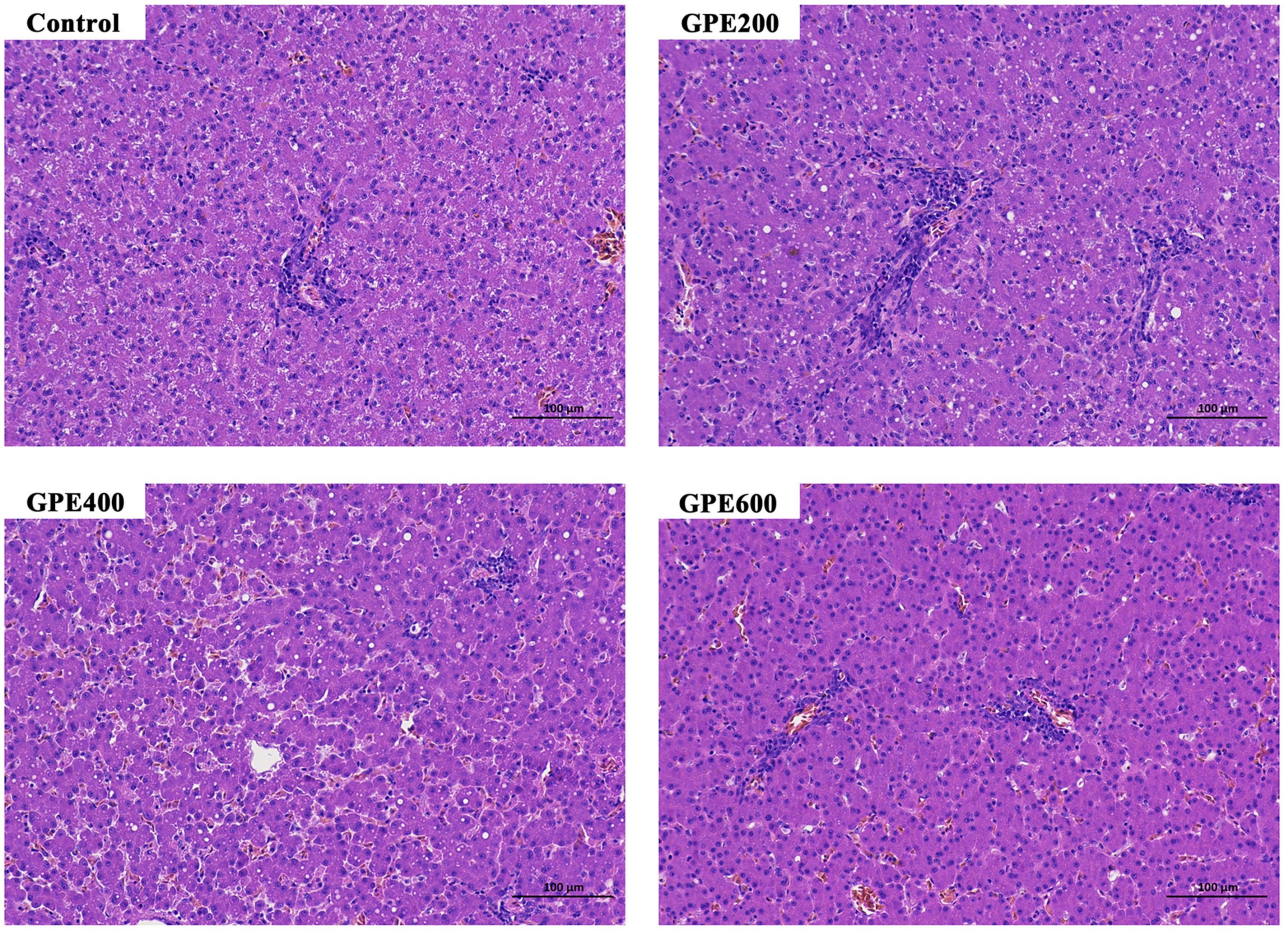
Effect of supplementation with GPE on histopathological alternations of liver tissue.

To further explore whether GPE exerted potential toxic effect on the liver, the histological architecture of the liver was observed by microscope following H&E staining. As showcased in Figure 2, in the control group, the structure of liver tissues was intact with regular arrangement of hepatocyte cells. However, in parallel to the control group, dietary inclusion of GPE did not result in any noticeable morphological changes in hepatocytes. This finding suggested that GPE supplementation had no potential cytotoxic effects on the liver, highlighting its suitability and safely as a feed additive.

### 3.8 Meat quality

To estimate the effect of GPE supplementation on muscle characteristics and meat quality traits, the related parameters of meat quality (pH, color, water loss rate, and shear force) were measured. The effect of dietary GPE on the meat quality of ducks were presented in Table 8. Dietary supplementation with GPE showed no effect on the pH of breast or leg muscles. Additionally, no significant differences in meat color values were detected, including a^*^, b^*^, and L^*^. Notably, in parallel to the control group, dietary supplementation with GPE resulted in a substantial depression of water loss rate and shear force. These results indicated that diets with GPE supplementation could effectively improve shear force and reduce water loss rate, leading to an overall improvement in meat quality.

**Table 8:** Effect of GPE supplementation on meat quality of meat ducks.

Items^1^	Diets				SEM	*P*
	CON	GPE200	GPE400	GPE600		
Breast muscle pH	5.73	5.74	5.74	5.78	0.02	0.887
Leg muscle pH	6.72	6.70	6.77	6.82	0.04	0.685
L^*^	39.39	41.03	40.50	40.63	0.48	0.672
a^*^	16.24	16.26	16.91	16.94	0.39	0.868
b^*^	3.89	4.03	5.03	4.06	0.24	0.304
Water loss rate (%)	34.99^a^	30.71^b^	30.85^b^	30.89^b^	0.67	0.045
Shear force (kgf)	5.17^a^	4.67^b^	4.52^b^	4.50^b^	0.09	0.014
IMP (mg/g)	1.37	1.20	1.13	1.29	0.62	0.582

Values of “L^*^,” “a^*^,” “b^*^” represent brightness, redness and yellowness of meat surface. SEM, standard error (SE) of means.

1 Different lowercase letters represent significant differences (*p* < 0.05) and data are the mean of 6 replicates with 2 ducks each replicate.

### 3.9 The IMP content of breast muscle

As shown in Table 8, the IMP content in the breast muscle meat of the GPE-treated groups was not significantly different from that of the control group at 70 days, indicating that GPE supplementation has no impact on modulating IMP level.

### 3.10 Chemical composition of the duck breast muscle

As displayed in Table 9, there was no noticeable effect of GPE on protein and fat (*p* > 0.05) content in the duck breast muscle. However, the inclusion of GPE (600 mg/kg) apparently improved the content of moisture in duck breast muscle. These findings suggested that GPE supplementation had no significant effect on the content of protein and fat but increased moisture, ultimately improving meat quality to some extent.

**Table 9:** Effect of GPE supplementation on protein, fat, moisture content and antioxidant capacity of duck breast muscle.

Items^1^	Diets				SEM	*p*
	CON	GPE200	GPE400	GPE600		
Moisture (%)	69.17^b^	68.74^b^	67.51^c^	70.47^a^	1.65	<0.001
Fat (%)	2.51	2.52	2.54	2.49	0.18	0.956
Protein (%)	20.07	20.21	18.78	19.21	0.49	0.715
TC (mmol/g)	0.25	0.24	0.25	0.26	0.01	0.495
MDA (nmol/mg prot)	0.47	0.51	0.57	0.53	0.15	0.102

SEM, standard error (SE) of means.

1 Different lowercase letters represent significant differences (*p* < 0.05) and data are the mean of 6 replicates with 2 ducks each replicate.

### 3.11 Antioxidant capacity of the duck breast muscle

As depicted in Table 9, in contrast to the control group, the levels of MDA and TC did not show any significant variations after GPE supplementation. These results demonstrated that GPE inclusion had no significant impact on improving antioxidant capacity of breast muscle or modulating its TC level.

## 4 Discussion

Over the last several decades, therapeutic practices in global animal production have heavily relied on synthetic antibiotics, with approximately 70% usage (22, 23). This widespread reliance is primarily due to their remarkable efficacy in boosting antioxidative defenses, mitigating inflammatory responses, and improving both overall health status and production performance (24). However, with increasing concern about the negative impact of antibiotic use aspects on public health, many countries have gradually been banning the use of antibiotics in animal husbandry production (25–27). Despite strict restrictions on the use of antibiotics, maintaining poultry health remains a formidable challenge in livestock and poultry production. In efforts to address this issue, researchers have gradually turned their attention to natural plants which could enhance immunity and antioxidant capacity of animals and birds (28). Therefore, it is imperative to search for efficient, eco-friendly, and safe feed additives that can enhance animals’ production potential, improve body health, and serve as a viable alternative to antibiotics in feed.

Through a long history of clinical practice, TCM has developed a unique theoretical framework and abundant clinical experience aimed at enhancing the health of both humans and animals (29). In recent, with the ban on antibiotics, TCM has been widely used in livestock farming due to its high efficacy and low toxicity (30). Not only these, new natural compounds derived from TCM have been also increasingly used as feed additives (31, 32). Furthermore, many lines of evidence have indicated that dietary supplementation with TCM has a positive effect on improving animal health, providing a promising and effective alternative to antibiotics in livestock production (33). A large body of animal experiment results also confirmed that dietary supplementation with TCM has more advantages in promoting livestock growth and improving disease resistance in comparison to conventional feed additives (34). Therefore, more and more researchers are also aware of the vital role of TCM in livestock farming (35). It can thus be stated that TCM has the potential to be a new feed additive to improve livestock productivity and health status.

GP is a traditional folk herbal medicine widely used in China, which is clinically used for the treatment of eruptive fevers and inflammatory-related diseases. It contained a series of biologically active components, including flavonoids, polyphenols, saponin, tannin and terpenoid (18, 36). Research has indicated that GP leaf extract could enhance hepatic antioxidation capacity and decrease pro-inflammatory factor levels in mice, effectively mitigating cadmium-induced liver toxicity (37). Furthermore, other investigators have found that dietary supplementation of GP or GP-containing herbal prescriptions could reduce the egg yolk cholesterol, suppress harmful excreta microflora and improve layers performance, which was ascribed to the bioactive compounds in GP (38). From this, GPE has the potential to be a new feed additive to improve livestock farming applications. Therefore, to delve deeper into whether the potential effects of GPE on ducks are associated with immune enhancement and improved antioxidant functions, we measured immune and anti-oxidation-related parameters.

Serum biochemical parameters provide valuable information for evaluating the health status of ducks and reflect many metabolic alternations of organs and tissues. ALT, AST and ALP are the commonly used indicators for assessing liver function. Once liver was injured, hepatocytes were destroyed contributing to the sharply increased serum aminotransferases (39). Urea nitrogen is the primary metabolite of protein and amino acid, which reflects the utilization efficiency of nitrogen (40). Serum total protein and ALB could reflect the metabolic status of protein in the body to a certain extent (41). TC is an important index that could indirectly reflect the ability of hepatic lipid synthesis (42). Additionally, TG is the recognized indicator, which is used to further evaluate the function of hepatic lipid metabolism (43).

In this work, there is no statistically significant difference in ALT, AST, and ALP levels between the control group and treatment groups, indicating dietary supplementation of GPE did not exert any toxic effect on the liver. Notably, dietary supplementation of GPE dramatically reduced the levels of total protein and BUN, suggesting the utilization efficiency of nitrogen was dramatically increased after GPE supplementation. Furthermore, dietary supplementation of GPE improved hepatic lipid metabolism, as evidenced by the reduced levels of TC and TG. Concordant with our results, Murugaiyah et al. reported that serum TC level was decreased in chemical and high fat diet (HFD)-induced hyperlipidemic rats treated with GPE (44). Hence, these results illustrated that dietary supplementation with GPE could modulate hepatic lipid metabolism and improve nitrogen utilization efficiency in ducks.

Accumulated lines of evidence have indicated that immunity is intimately correlated with livestock health (45). The immune system guards the body against foreign substances and protects it from invasion by pathogenic organisms. As the important regulators of immune function, Ig is a class of specific active proteins that can be converted into antibodies by antigen induction, such as IgM, IgG, IgA and so on (46). The immunoglobulin activities (IgA, IgM, and IgG) in the serum are important indicators to estimate the non-specific immunity status of the animal. Immunoglobulin A (IgA), an important serum immunoglobulin, plays a crucial role in the immune defense of mucosal surfaces (47). Additionally, when the body is exposed to an external stimulus, the immune system is primed, triggering the release of several inflammatory factors, such as IL-1β and IL-6 (48). In the present experiment, dietary inclusion of GPE could dramatically elevate the contents of IgM and IgG at day 70 of age in compared to the control group. The IL-2 level was considerably inhibited by dietary GP supplementation. Curiously, treatment with GPE has no evident difference in IL-6 and IL-1β activities. Consistent with our observations, Wu et al. found that dietary supplementation with flavonoids from bamboo leaf could elevate the serum IgM activity in comparison to the control group, which might be attributed to the potent immunomodulatory functions of flavonoids (49). Additionally, Huang et al. also noted that supplementation with flavonoid-rich Fenugreek extract had a positive effect on modulating immunity in broiler (50). The enhanced immunity may result from GPE’S ability to stimulate immune cells to release immune factors, thereby attenuating inflammation (51).

In addition to modulating immunity function, numerous studies have revealed that oxidative stress was also a crucial contributor to inferior growth performance of ducks (52). To further elucidate whether the potential effect of GPE was intimately associated with its antioxidant function, the oxidative stress-related indicators were determined. MDA, as the final product of lipid peroxidation, is widely considered a reliable marker reflecting the degree of oxidative damage (53). GSH-Px are the key antioxidant enzymes that can scavenge ROS generated from oxidant stress (54). SOD is a pivotal endogenous antioxidant enzyme that acts as a component of first-line defense against oxidative damage. It catalyzes the dismutation of superoxide anion, forming hydrogen peroxide and molecular oxygen (55). T-AOC was the main parameter to measure the total antioxidant level of the enzymatic and nonenzymatic systems (56). In the present experiment, our results indicated that dietary supplementation with GPE could dramatically elevate the activity of T-AOC and reduce MDA content in serum, which might largely be attributed to its primary bioactive ingredient, polyphenolic. Similar to our finding, Ao et al. demonstrated that grape seeds were also richer in polyphenolic compounds and confirmed that supplementation with grape seeds dramatically enhanced antioxidative activity by improving T-AOC in broilers (57). The excellent antioxidant capacity might be closely associated with polyphenol, flavonoids in GPE, all of which exhibit potent antioxidant and free radical scavenging activities (36).

Carcass characteristics have been recognized as crucial response parameters to assess dietary energy and amino acid status in livestock dietary, such as yields of breast muscle, thigh muscle, and abdominal fat (58). Our findings suggested that no significant variance in dressing yield was observed between the control group and the three treatment groups. Additionally, there was no statistically significant difference in dressing percentage, semi-eviscerated yield, eviscerated yield, breast muscle yield and abdominal fat yield between the various treated groups. In agreement with our results, Ma et al. reported that there were no pronounced effects on dressing percentage, eviscerated yield, breast and thigh percentage after supplementation with flavonoids derived from sea buckthorn fruits (59). Altogether, these findings indicated that dietary supplementation with GPE did not show any substantial effect on the carcass characteristics.

The organ indexes are important indicators, which could partially reflect the health status of the body. When the body is challenged by an external stimulus, the internal organs of the body will undergo corresponding changes. Among them, the spleen is an important immune organ, which protects the host from microbial infections and mechanical injuries. The liver is a central organ for lipid metabolism and plays a vital role in detoxification. The lung is a particularly sensitive organ, which defenses the body from external stimulus. Our present results showed that dietary supplementation of GPE had no implications on the various organs, such as heart, liver, kidney, spleen, gizzard and glandular stomach. These data demonstrated that dietary supplementation with GPE did not exert toxic side effects on these organs, confirming that dietary supplementation with GPE is a safe strategy for improving animal health.

As we all know, in addition to their impact on the growth and health of livestock, abnormal oxidative stress and immunity influence have also a negative effect on the quality of livestock meat (60). Meat quality is an essential element that influences consumer’s purchasing decisions. As the consumer’s demand for meat quality is increasing, improving the quality of meat becomes a highly effective approach to boost the consumer’s purchase desire (61). Recently, more and more traditional Chinese medicine, such as *Astragalus mongholicus Bunge* and *Largehead Atractylodes* Rh, have been confirmed to exhibit a favorable effect on the improvement of meat quality (33, 62). Therefore, to investigate whether the potential effect of GPE was closely linked to improving meat quality, the meat quality relevant parameters were measured. In this work, dietary supplementation with GPE apparently improved the moisture content of breast muscle, while it had no notable variation in protein, fat and IMP contents of the breast muscle. In keeping with our observation, Ahmad et al. found that *β*-GOS and methionine co-supplementation did not show any promising effects on meat quality in broilers (63). Furthermore, dietary supplementation of GPE could apparently reduce the shear force and the water loss rate. In addition, no marked difference in breast muscle pH, leg muscle pH and breast muscle color were observed in any of the experimental groups. As we all know, pH value is a vital reference for judging the change of acidity in the process of muscle tissue fermentation and speed of fermentation after slaughtering, which has a substantial impact on meat quality. Therefore, maintaining a stable pH is essential for the proper maturation of muscles. Recent studies have demonstrated that a rapid pH reduction causes denaturation of myofibrillar proteins, leading to poor water-holding capacity and deteriorated drip loss (64). In this investigation, the reduced water loss in the breast muscle may be attributed to a stable pH value. The above results suggested that dietary supplementation with GPE prominently enhanced the tenderness and water-holding capacity of duck meat, leading to a notable improvement in its overall quality. Consistent with our results, Jin et al. reported that dietary supplementation with curcumin influenced the color of leg muscle while enhanced a prominent trend toward improving the water-holding capacity in breast muscle of meat ducks (65). The integrity of the cell membrane is a crucial factor that influences the water-holding capacity and tenderness of meat. Enhancement in meat quality may be caused by the fact the stability and integrity of the cell membrane were changed due to the improved antioxidant capacity of meat (66).

Taken together, our study first demonstrated that there was no significant effect on growth performance after dietary supplementation with GPE. Noteworthy, dietary supplementation with GPE could enhance immunity and regulate oxidative stress. Moreover, supplementation of GPE could improve meat quality, which was primarily manifested by improvements in the shear force and water loss rate. It might offer a strong rationale for developing and utilizing GPE and GP-containing traditional Chinese herbal prescriptions as a novel feed additive. Furthermore, our discovery would have far-reaching implications for expanding the potential utility of GPE.

Apart from these strengths, there are several additional limitations to our study. Firstly, despite GPE exhibiting favorable effect on ducks, it is not clearly established which bioactive ingredients are associated with its excellent modulation effect. Secondly, the study is currently restricted to ducks only and has not been extended to other conventional animals. Thirdly, our study only focused on the modulation effect of GPE in ducks, which neglected the dynamic process of GPE *in vivo*. To address these limitations, the detailed bioactive ingredients still need a deeper investigation. In addition, the specific regulatory mechanism of GPE as immunomodulators and antioxidants deserves to be further investigated. Furthermore, more different kinds of animals were required to investigate its potential effect before widespread application. By explicitly noting these constricts, we aim to elucidate which active components exhibited the best modulation effect on ducks. Besides, we also encourage ongoing research to investigate the dynamic process of GPE *in vivo* for a clearer understanding of the underlying molecular mechanisms.

## 5 Conclusion

In conclusion, this study showed that dietary supplementation with GPE could increase serum total antioxidant capacity, regulate immunity function and improve meat quality to some extent in meat ducks. These results can provide further evidence using GPE as a feed additive in meat duck production. The recommended optimal GPE level in the diet of meat ducks is 600 mg/kg according to the results in this study.

## Data Availability

The datasets presented in this study can be found in online repositories. The names of the repository/repositories and accession number(s) can be found in the article/supplementary material.
